# Supplementary material for: Allosteric effects of the coupling cation in melibiose transporter MelB
Source: eLife. 2026 Jan 28;14:RP108335. doi: 10.7554/eLife.108335 (PMC12851581; doi:10.7554/eLife.108335)
Supplement: Supplementary file 1. [file elife-108335-supp1.docx]

**Supplementary File 1.** **Crystallographic Data collection, phase, and refinement statistics**

| **Data collection** | D59C MelB_St_ complexed with α-galactosides | | | |
| --- | --- | --- | --- | --- |
|  | α-NPG  [PDB ID, 9OLD] | Melibiose  [PDB ID, 9OLI] | α-Methyl galactoside  [PDB ID, 9OLR] | Raffinose  [PDB ID, 9OLP] |
|  | ALS 5.0.2  0.9795 | ALS 5.0.2  0.9795 | ALS 5.0.2  0.9795 | ALS 5.0.1  0.97 |
| Space group | P 31 2 1 | P 31 2 1 | P 31 2 1 | P 31 2 1 |
| Cell dimensions |  |  |  |  |
| *a*, *b*, *c* (Å) | 126.9 126.9 104.4 | 126.4 126.4 103.5 | 127.496 127.496 106.039 | 127.1 127.1 104.9 |
| α, β, γ (°) | 90 90 120 | 90 90 120 | 90 90 120 | 90 90 120 |
| Resolution (Å) | 20 - 2.60 | 20 - 3.05 | 20 - 3.68 | 20 - 3.40 |
| *R*_meas_ | 0.072 (3.299) | 0.067 (2.064) | 0.145 (0.508) | 0.339 (1.297) |
| *I* / σ*I* | 16.5 (0.9) | 11.8 (0.8) | 8.4 (4.4) | 7.9 / 3.1 |
| CC(1/2) | 1.00 (0.40) | 1.00 (0.26) | 1.00 (0.93) | 1.00 (0.873) |
| Completeness (%) | 98.6 (98.4) | 99.50 (100) | 99.8 (93.3) | 99.5 (100) |
| Redundancy | 9.7 (10.3) | 5.7 (5.7) | 9.2 (8.8) | 18.3 (17.5) |
|  |  |  |  |  |
| **Refinement** |  |  |  |  |
| Resolution (Å) | 20 - 2.60  (2.63 - 2.60) | 20 - 3.05  (3.13 - 3.05) | 20 - 3.68 (4.05 - 3.68) | 20 - 3.40  (3.54 - 3.40) |
| No. reflections | 29768 (1617) | 18439 (1410) | 10983 (2671) | 13237 (1424) |
| *R*_work_ / *R*_free_ | 0.256/0.287 | 0.255 / 0.294 | 0.297 / 0.330 | 0.316 / 0.329 |
| No. atoms | 3584 | 3540 | 3570 | 3581 |
| Protein | 3542 | 3515 | 3557 | 3547 |
| Ligand/ion | 37 | 23 | 13 | 34 |
| Water | 5 | 2 | 0 | 0 |
| *B*-factors | 121.88 | 135.70 | 116.76 | 113.7 |
| Protein | 121.93 | 135.64 | 116.78 | 113.4 |
| Ligand/ion | 121.31 | 146.75 | 109.95 | 109.6 |
| Water | 95.54 | 109.73 | / | / |
|  |  |  |  |  |
| R.m.s. deviations |  |  |  |  |
| Bond lengths (Å) | 0.003 | 0.003 | 0.002 | 0.002 |
| Bond angles (°) | 0.573 | 0.545 | 0.486 | 0.565 |

A single crystal was used for all structures. Values in parentheses are for highest-resolution shell.

**Table S2. HDX reaction, labeling details, and** **statistics**

|  | ΔD_Mel - Apo_ | ΔD_Na(+) - Apo_ | ΔD_Na(+)Mel – Apo_# |
| --- | --- | --- | --- |
| Samples measured | Test-1: WT MelB_St_ (Apo);  Test-2: WT MelB_St_ with 50 mM melibiose. | Test-3: WT MelB_St_ (Apo);  Test-4: WT MelB_St_  with 150 mM Na^+^. | Test-5: WT MelB_St_  with 50 mM melibiose and 150 mM Na^+^. |
| HX reaction buffer | 25 mM Tris-HCl, pD 7.5, 150 mM NaCl, 10% Glycerol, and 0.01% DDM | 25 mM Tris-HCl, pD 7.5, 150 mM NaCl, 10% Glycerol, and 0.01% DDM | 25 mM Tris-HCl, pD 7.5, 150 mM NaCl, 10% Glycerol, and 0.01% DDM |
| Reaction temperature (°C) | 20 | 20 | 20 |
| HX time course (s) | 0, 30, 300, 3000 | 0, 30, 300, 3000 | 0, 30, 300, 3000 |
| Number of peptides | 150 | 152 | 150 |
| Sequence coverage by labeling | 87.47 | 86.62 | 87.47 |
| Mean peptide length | 8.4 | 7.9 | 8.0 |
| Average redundancy | 3.7 | 3.5 | 3.5 |
| Replicates (technical) | 3 | 3 | 3 |
| \|ΔD\| (Da) | 0.186 | 0.224 | 0.175 |
| Back exchange rate | Not appliable | Not appliable | Not appliable |
| Number of non-covered positions | 59 | 63 | 59 |
| Threshold | ± 0.186 | ± 0.224 | ± 0.175 |
| Number of overlapping peptides with significant ΔD  > \| Threshold \| and P < 0.05 | 27 | 21 | 30 |
| Number of covered residues with significant ΔD  > \| Threshold \| and P < 0.05 | 153 | 122 | 133 |
| Number of covered residues with insignificant ΔD  <\| Threshold \| and P > 0.05 | 237 | 264 | 257 |

# Test-1 data of Apo MelB_St_ was used for comparison and calculation.

**Table S3.** **Relative deuterium uptake and uncovered positions of the Apo MelB_St_**

| Region | | Fragment  (#residues) | HDX coverage  (# residues) | Averaged D%^*^  (n = 2) | 59 of uncovered fragments  (#residues) | Sequence |
| --- | --- | --- | --- | --- | --- | --- |
| H-I | 4 - 38 (35) | | 2 - 35 (34) | 8.531 ± 0.865^#^ | 0 |  |
| H-II | 40 - 68 (29) | | 36 - 77 (42) | 1.736 ± 0.345 | 0 |  |
| H-III | 76 - 100 (25) | | 76 - 100 (25) | 1.465 ± 0.037 | Position 92 (1) | F^92^ |
| H-IV | 103 - 135 (33) | | 99 - 138 (40) | 2.083 ± 0.004 | Positions 111-119 (9) | V^111^TYILWGMT^119^ |
| H-V | 137 - 171 (35) | | 137 - 169 (32) | 7.069 ± 0.022^#^ | 0 |  |
| H-VI | 174 - 201 (28) | | 170 - 205 (36) | 1.627 ± 0.205 | Positions 181-183 (3) | F^182^TL |
| Loop_6-7_ | 202 - 230 (29) | | 209 - 233 (25) | 4.955 ± 0.291 | Positions 206-208 (3) | S^206^SD |
| H-VII | 231 - 261 (31) | | 227 - 263 (37) | 1.767 ± 0.136 | 0 |  |
| H-VIII | 266 - 285 (20) | | 264 - 283 (20) | 3.587 ± 0.218 | Positions 272-273 (2) | L^272^S^273^ |
| Loop_8-9_ | 286 - 295 (10) | | 282 - 298 (17) | 10.876 ± 0.189 | 0 |  |
| H-IX | 296 - 319 (24) | | 296 - 314 (18) | 1.921 ± 0.037 | Positions 303-308, 315-316 (8) | S^303^VMPVL^308^  A^315^M^316^ |
| Loop_9-10_ | 320-323 (4) | | 317-326 (10) | 6.475 ± 1.165 | 0 |  |
| H-X^ | 323 - 360 (38) | | 342 - 360 (18)^##^ | 1.992 ± 0.388 | Positions 327-341 and 345-348 (19) | I^327^VAAGIFLNIGTALF^341^  Q^345^VIM^348^ |
| Loop_10-11_ | 361 – 365 (6) | | 364 - 368 (5) | 19.94 | Positions 360-363 (4) | L^360^NIR^363^ |
| H-XI | 366 – 395 (30) | | 369 - 386 (19) | 2.207 ± 0.007 | Positions 387-393 (7) | I^387^ALVLGL^393^ |
| Loop_11-12_^ | 396 – 403 (8) | | 394 - 406 (13)^##^ | 3.581 ± 2.046 | 0 |  |
| H-XII | 404 – 432 (31) | | 406 - 435 (30) | 1.395 ± 0.184 | 0 |  |
| C-term Tail^ | 433 – 476 (38) | | 439 - 470 (32)^##^ | 40.890 ± 3.507 | Positions 436 – 438 (3) | N^436^GD^438^ |

^*^, Average from two dataset of apo state of the mean values of relative deuterium uptake (D%) across all time points (30 sec, 300 sec, and 3000 sec) of all covered peptides.

^, The uncovered residues from the dataset of ΔD_Na(+)-Apo_ are different, as listed here: H-X (Positions 327-348; 22 residues); Loop_10-11_ (Positions 360-363; 3 residues); Loop_12-CTH_ (Positions 436-440; 5 residues). The total number of the uncovered positions for Na+ vs. apo dataset is 63.

^#^, Averaged D% of helix I vs helix II, P =0.01; D% of helix V vs helix II, P =0.001. Unpaired t-test was applied for the data after log transformation.

^##^, the starting or sending positions between the two datasets were slightly different, with positions 349-360, 394-407 and 441-470 presented in the apo date from the ΔD_Na(+)-Apo_ dataset, respectively.

**Table S4. HDX results at the sugar- and Na^+^-binding pockets**

|  | **Mel (0.1857^*^)** | | | | | | **Na^+^ (0.224)** | | | | | | | | **Mel with Na^+^ (0.1754)** | | | | | | | |
| --- | --- | --- | --- | --- | --- | --- | --- | --- | --- | --- | --- | --- | --- | --- | --- | --- | --- | --- | --- | --- | --- | --- |
|  | Peptides | Data  (P<0.05) | Protection^^^ | | Peptides | | | Data  (P<0.05)^$^ | | | Protection^^^ | | | | Peptides | | | Data  (P<0.05)^$^ | | Protection^^^ | | |
| **Sugar-binding residues** | | | | | | | | | | | | | | | | | | | | | | |
| K18 | 4 | 2 | 1 | | | 3 | | | 2 | | | 1 | | | 3 | | | | 3 | 2 | | |
| D19 | 3 | 2 | 1 | | | 4 | | | 3 | | | 2 | | | 3 | | | | 3 | 2 | | |
| I22 | 1 | 1 | 1 | | | 4 | | | 4 | | | 2 | | | 1 | | | | 1 | 1 | | |
| Y26 | 3 | 1 | 1 | | | 3 | | | 3 | | | 1 | | | 3 | | | | 5 | 1 | | |
| Y120 | 1 | 1 | 0 | | | 1 | | | 0 | | | / | | | 1 | | | | 2 | 0 | | |
| D124 | 2 | 1 | 0 | | | 2 | | | 3 | | | 0 | | | 2 | | | | 2 | 0 | | |
| W128 | 2 | 0 | / | | | 3 | | | 5 | | | 0 | | | 3 | | | | 3 | 0 | | |
| R149 | 9 | 17 | 8 | | | 9 | | | 17 | | | 7 | | | 9 | | | | 27 | 18 | | |
| A152 | 1 | 1 | 0 | | | 1 | | | 0 | | | 0 | | | 1 | | | | 2 | 1 | | |
| W342 | 1 | 0 | / | | | 0 | | | / | | | / | | | 1 | | | | 0 | / | | |
| Q372 | 1 | 2 | 0 | | | 1 | | | 3 | | | 1 | | | 1 | | | | 3 | 1 | | |
| T373 | 2 | 2 | 0 | | | 2 | | | 3 | | | 1 | | | 2 | | | | 3 | 1 | | |
| V376 | 2 | 0 | / | | | 3 | | | 2 | | | 0 | | | 2 | | | | 1 | 0 | | |
| K377 | 2 | 0 | / | | | 3 | | | 2 | | | 0 | | | 2 | | | | 1 | 0 | | |
|  |  |  | |  | | |  | | |  | | |  | |  |  | | | | |  | |
| **Cation-binding residues** | | | | | | | | | | | | | | | | | | | | | |  |
| 55 | 3 | 2 | 0 | | | 3 | | | 5 | | | 0 | | 3 | | | 2 | | | 0 | | |
| 58 | 3 | 2 | 0 | | | 3 | | | 5 | | | 0 | | 3 | | | 2 | | | 0 | | |
| 59 | 4 | 2 | 0 | | | 4 | | | 5 | | | 0 | | 4 | | | 2 | | | 0 | | |
| 121 | 2 | 1 | 0 | | | 2 | | | 1 | | | 0 | | 2 | | | 3 | | | 0 | | |

**^*^** Threshold values.

^^^ P < 0.05 and D > | threshold | at any time point.

**Table S5. Structure information**

| **PDB ID**  **(Ligand)** | **9OLD**  (**α-NPG)** | **9OLI**  **(Melibiose)** | **9OLR**  **(α-MG)** | **9OLP**  **(Raffinose)** |
| --- | --- | --- | --- | --- |
| Resolved positions | 2-255 | 2-253 | 2-254 | 2-254 |
| Missing side chains | Lys221  Lys291  His322  Arg431  Lys450  Lys453 | Arg70  Arg199  Val261  Leu267  Lys291  Asp320  His322  Leu334  Asn399  Lys450 | Lys291 | Arg70  His322  Leu447 |
| Ramachandran  Favored (%)  Outliers  Clash scores | 97.57  0.00  1.25 | 96.89  0.22  1.40 | 94.46  1.33  2.9 | 94.46  0.89  4.42 |

**Table S6.** **MD simulations of Wat-1 occupancy in sugar-bound MelB_St_ with or without Na^+^**

| **System** | **Replica** | **Occupancy** |
| --- | --- | --- |
| **Sugar + Na^+^** | 1 | 97.49% |
|  | 2 | 92.16% |
|  | 3 | 98.86% |
|  | 4 | 96.48% |
|  | 5 | 99.21% |
|  | **Average** | **96.84% (**$\pm$**2.83%^)** |
| **Sugar Only** | 1 | 99.25% |
|  | 2 | 94.64% |
|  | 3 | 99.56% |
|  | 4 | 96.42% |
|  | 5 | 94.31% |
|  | **Average** | **96.84% (**$\pm$**2.48%)** |

**^, SD**
