# Supplementary material for: Allosteric effects of the coupling cation in melibiose transporter MelB
Source: eLife. 2026 Jan 28;14:RP108335. doi: 10.7554/eLife.108335 (PMC12851581; doi:10.7554/eLife.108335)
Supplement: Supplementary file 2. [file elife-108335-supp2.docx]

**Supplementary File 2. HDX reaction, labeling details, and** **statistics**

|  | ΔD_Mel - Apo_ | ΔD_Na(+) - Apo_ | ΔD_Na(+)Mel – Apo_# |
| --- | --- | --- | --- |
| Samples measured | Test-1: WT MelB_St_ (Apo);  Test-2: WT MelB_St_ with 50 mM melibiose. | Test-3: WT MelB_St_ (Apo);  Test-4: WT MelB_St_  with 150 mM Na^+^. | Test-5: WT MelB_St_  with 50 mM melibiose and 150 mM Na^+^. |
| HX reaction buffer | 25 mM Tris-HCl, pD 7.5, 150 mM NaCl, 10% Glycerol, and 0.01% DDM | 25 mM Tris-HCl, pD 7.5, 150 mM NaCl, 10% Glycerol, and 0.01% DDM | 25 mM Tris-HCl, pD 7.5, 150 mM NaCl, 10% Glycerol, and 0.01% DDM |
| Reaction temperature (°C) | 20 | 20 | 20 |
| HX time course (s) | 0, 30, 300, 3000 | 0, 30, 300, 3000 | 0, 30, 300, 3000 |
| Number of peptides | 150 | 152 | 150 |
| Sequence coverage by labeling | 87.47 | 86.62 | 87.47 |
| Mean peptide length | 8.4 | 7.9 | 8.0 |
| Average redundancy | 3.7 | 3.5 | 3.5 |
| Replicates (technical) | 3 | 3 | 3 |
| \|ΔD\| (Da) | 0.186 | 0.224 | 0.175 |
| Back exchange rate | Not appliable | Not appliable | Not appliable |
| Number of non-covered positions | 59 | 63 | 59 |
| Threshold | ± 0.186 | ± 0.224 | ± 0.175 |
| Number of overlapping peptides with significant ΔD  > \| Threshold \| and P < 0.05 | 27 | 21 | 30 |
| Number of covered residues with significant ΔD  > \| Threshold \| and P < 0.05 | 153 | 122 | 133 |
| Number of covered residues with insignificant ΔD  <\| Threshold \| and P > 0.05 | 237 | 264 | 257 |

# Test-1 data of Apo MelB_St_ was used for comparison and calculation.
