# Supplementary material for: Allosteric effects of the coupling cation in melibiose transporter MelB
Source: eLife. 2026 Jan 28;14:RP108335. doi: 10.7554/eLife.108335 (PMC12851581; doi:10.7554/eLife.108335)
Supplement: Supplementary file 4. [file elife-108335-supp4.docx]

**Supplementary File 4. HDX results at the sugar- and Na^+^-binding pockets**

|  | **Mel (0.1857^*^)** | | | | | | **Na^+^ (0.224)** | | | | | | | | **Mel with Na^+^ (0.1754)** | | | | | | | |
| --- | --- | --- | --- | --- | --- | --- | --- | --- | --- | --- | --- | --- | --- | --- | --- | --- | --- | --- | --- | --- | --- | --- |
|  | Peptides | Data  (P<0.05) | Protection^^^ | | Peptides | | | Data  (P<0.05)^$^ | | | Protection^^^ | | | | Peptides | | | Data  (P<0.05)^$^ | | Protection^^^ | | |
| **Sugar-binding residues** | | | | | | | | | | | | | | | | | | | | | | |
| K18 | 4 | 2 | 1 | | | 3 | | | 2 | | | 1 | | | 3 | | | | 3 | 2 | | |
| D19 | 3 | 2 | 1 | | | 4 | | | 3 | | | 2 | | | 3 | | | | 3 | 2 | | |
| I22 | 1 | 1 | 1 | | | 4 | | | 4 | | | 2 | | | 1 | | | | 1 | 1 | | |
| Y26 | 3 | 1 | 1 | | | 3 | | | 3 | | | 1 | | | 3 | | | | 5 | 1 | | |
| Y120 | 1 | 1 | 0 | | | 1 | | | 0 | | | / | | | 1 | | | | 2 | 0 | | |
| D124 | 2 | 1 | 0 | | | 2 | | | 3 | | | 0 | | | 2 | | | | 2 | 0 | | |
| W128 | 2 | 0 | / | | | 3 | | | 5 | | | 0 | | | 3 | | | | 3 | 0 | | |
| R149 | 9 | 17 | 8 | | | 9 | | | 17 | | | 7 | | | 9 | | | | 27 | 18 | | |
| A152 | 1 | 1 | 0 | | | 1 | | | 0 | | | 0 | | | 1 | | | | 2 | 1 | | |
| W342 | 1 | 0 | / | | | 0 | | | / | | | / | | | 1 | | | | 0 | / | | |
| Q372 | 1 | 2 | 0 | | | 1 | | | 3 | | | 1 | | | 1 | | | | 3 | 1 | | |
| T373 | 2 | 2 | 0 | | | 2 | | | 3 | | | 1 | | | 2 | | | | 3 | 1 | | |
| V376 | 2 | 0 | / | | | 3 | | | 2 | | | 0 | | | 2 | | | | 1 | 0 | | |
| K377 | 2 | 0 | / | | | 3 | | | 2 | | | 0 | | | 2 | | | | 1 | 0 | | |
|  |  |  | |  | | |  | | |  | | |  | |  |  | | | | |  | |
| **Cation-binding residues** | | | | | | | | | | | | | | | | | | | | | |  |
| 55 | 3 | 2 | 0 | | | 3 | | | 5 | | | 0 | | 3 | | | 2 | | | 0 | | |
| 58 | 3 | 2 | 0 | | | 3 | | | 5 | | | 0 | | 3 | | | 2 | | | 0 | | |
| 59 | 4 | 2 | 0 | | | 4 | | | 5 | | | 0 | | 4 | | | 2 | | | 0 | | |
| 121 | 2 | 1 | 0 | | | 2 | | | 1 | | | 0 | | 2 | | | 3 | | | 0 | | |

**^*^** Threshold values.

^^^ P < 0.05 and D > | threshold | at any time point.
