# Supplementary material for: Allosteric effects of the coupling cation in melibiose transporter MelB
Source: eLife. 2026 Jan 28;14:RP108335. doi: 10.7554/eLife.108335 (PMC12851581; doi:10.7554/eLife.108335)
Supplement: Supplementary file 5. [file elife-108335-supp5.docx]

**Supplementary File 5. Structure information**

| **PDB ID**  **(Ligand)** | **9OLD**  (**α-NPG)** | **9OLI**  **(Melibiose)** | **9OLR**  **(α-MG)** | **9OLP**  **(Raffinose)** |
| --- | --- | --- | --- | --- |
| Resolved positions | 2-255 | 2-253 | 2-254 | 2-254 |
| Missing side chains | Lys221  Lys291  His322  Arg431  Lys450  Lys453 | Arg70  Arg199  Val261  Leu267  Lys291  Asp320  His322  Leu334  Asn399  Lys450 | Lys291 | Arg70  His322  Leu447 |
| Ramachandran  Favored (%)  Outliers  Clash scores | 97.57  0.00  1.25 | 96.89  0.22  1.40 | 94.46  1.33  2.9 | 94.46  0.89  4.42 |
