# Supplementary material for: Allosteric effects of the coupling cation in melibiose transporter MelB
Source: eLife. 2026 Jan 28;14:RP108335. doi: 10.7554/eLife.108335 (PMC12851581; doi:10.7554/eLife.108335)
Supplement: Supplementary file 6. [file elife-108335-supp6.docx]

**Supplementary File 6.** MD simulations of Wat-1 occupancy in sugar-bound MelB_St_ with or without Na^+^

| **System** | **Replica** | **Occupancy** |
| --- | --- | --- |
| **Sugar + Na^+^** | 1 | 97.49% |
|  | 2 | 92.16% |
|  | 3 | 98.86% |
|  | 4 | 96.48% |
|  | 5 | 99.21% |
|  | **Average** | **96.84% (**$\pm$**2.83%^)** |
| **Sugar Only** | 1 | 99.25% |
|  | 2 | 94.64% |
|  | 3 | 99.56% |
|  | 4 | 96.42% |
|  | 5 | 94.31% |
|  | **Average** | **96.84% (**$\pm$**2.48%)** |

**^, SD**
